# Soybean balanced the growth and defense in response to SMV infection under different light intensities

**DOI:** 10.3389/fpls.2023.1150870

**Published:** 2023-04-19

**Authors:** Jing Shang, Lu-Ping Zhao, Xin-Miao Yang, Xiao-Li Qi, Jin-Feng Yu, Jun-Bo Du, Kai Li, Cheng-Shan He, Wen-Ming Wang, Wen-Yu Yang

**Affiliations:** ^1^ Sichuan Engineering Research Center for Crop Strip Intercropping System and College of Agronomy, Sichuan Agricultural University, Chengdu, China; ^2^ National Center for Soybean Improvement, National Key Laboratory for Crop Genetics and Germplasm Enhancement, Key Laboratory of Biology and Genetic Improvement of Soybean, Ministry of Agriculture, Nanjing Agricultural University, Nanjing, China; ^3^ State Key Laboratory of Crop Gene Exploration and Utilization in Southwest, Sichuan Agricultural University, Chengdu, China

**Keywords:** *Soybean mosaic virus*, light intensity, growth, defense, soybean

## Abstract

Light is essential for the growth and defense of soybean. It is not clear how soybeans adjust their defenses to different light environments with different cropping patterns. The mechanism of soybean response to *Soybean mosaic virus* (SMV) infection under different light intensities was analyzed by RNA-seq sequencing method. Enrichment analysis illustrated that most defense-related genes were down-regulated in the dark and the shade, and up-regulated under hard light and normal light. Soybean can resist SMV infection mainly by activating salicylic acid signaling pathway. Light is essential for activating salicylic acid defense signaling pathways. With the increase of light intensity, the oxidative damage of soybean leaves was aggravated, which promoted the infection of virus. When light was insufficient, the growth of soybean was weak, and the plant-pathogen interaction pathway, MAPK pathway and hormone defense pathway in infected soybean was inhibited. Under hard light, some defense genes in infected soybean were down-regulated to reduce the degree of oxidative damage. The expression of differentially expressed genes was verified by real-time fluorescence quantitative RT-PCR. In order to adapt to the change of light intensity, soybean balanced allocation of resources between growth and defense through a series regulation of gene expression. The results of this study will provide a theoretical basis for the research of SMV resistance in intercropping soybean.

## 1 Introduction

Intercropping system maximizes the productivity as well as resource utilization per unit of land. As explained by the biodiversity theory, the maize-soybean intercropping system is an ecological strategy to control or relieve diseases (Zhang et al., 2019). In intercropping system, soybean undergoes complex changes in light environment (Li et al., 2021). During the symbiotic period of soybean and corn, soybean was in the shade. After the corn is harvested, soybeans get plenty of light. *Soybean mosaic virus* (SMV) was the most important soybean virus, which resulted in soybean yield reduction. How the soybean precisely allocates its limited resources between growth and defense under different light intensities is critical to its survival. Recent studies suggest that there may be complex regulatory mechanisms in plants, with interactions between hormone-based signaling networks causing transcriptional changes that balance the growth and defense (Du et al., 2018).

Our previous studies have shown that light can induce the eruption of reactive oxygen species (Shang et al., 2019) and activate the defense against pathogenic microorganisms (Shang et al., 2011). At the same time, light promoted the improvement of plant photosynthetic efficiency, which was conducive to the growth and development of plants. When light is insufficient, the growth of intercropping soybeans is inhibited, and the resources allocated to defense are correspondingly reduced (Zhang et al., 2019). Rewiring of jasmonate and phytochrome B signaling uncouples plant growth-defense tradeoffs (Campos et al., 2016). Light and darkness affect not only the host’s defense reaction, but also the pathogenicity of the pathogen (Telli et al., 2020).

Many studies have shown that light regulates the plant defense against pathogenic agents mainly through salicylic acid and jasmonic acid pathways (Zhang et al., 2019). NPR1 gene is a key gene in the salicylic acid pathway (Cao et al., 1998). Overexpression of NPR1 gene increases plant sensitivity to light, thereby enhancing disease resistance of Arabidopsis, crops and tobacco (Shang et al., 2011). Salicylic acid regulates NPRs protein to regulate the plant immunity (Backer et al., 2015). The expression of PR1 gene regulated by NPR1 protein is also induced by light (Agrawal et al., 2000). The expression of PR1 gene was down-regulated in stress treatments such as hard light, drought and salt (Wu et al., 2020). The main mechanism of defense inhibition is the simultaneous down-regulation of jasmonic acid and salicylic acid signaling through a low proportion of red: far-red light. Jasmonic acid signaling is inhibited by altering the balance between DELLA and Jasmonate ZIM DOMAIN (JAZ) proteins. The discovery of the link between photoreceptors and defense signals reveals a new mechanism that controls the allocation decisions of key resources in plant canopies. The decreased expression of phyB gene reduces the accumulation of jasmonic acid and inhibits the synthesis of insect-resistant protein in plants (Pierik and Ballare, 2021). Plants that silenced phyB genes showed decreased resistance to fungi (Courbier et al., 2020). Silencing photosystem II-related genes can reduce tobacco resistance to *Carrot mosaic virus* (Manfre et al., 2011). PhyA and phyB genes regulate RPS2 and R/MIN1 genes to enhance resistance to Pseudomonas cloves (Griebel and Zeier, 2008; Genoud et al., 2010). PhyA, phyB and phyC jointly regulate rice blast resistance (Xie et al., 2011). R gene (HRT) plays a role in Arabidopsis thaliana resistance to turnip leaf virus (TCV) (Jeong et al., 2010). Our previous studies revealed that WRKY transcription factor family genes participated in the interaction of salicylic acid and jasmonic acid signaling pathways (Shang et al., 2011; Zhang et al., 2019). Previous studies have also shown that GbWRKY1 negatively regulates the resistance of cotton to Boea cinerea through JA signaling pathway. OsWRKY13 regulates the expression of JA and SA upstream and downstream genes, and is involved in rice disease resistance.

Under the condition of limited resources, intercropping soybean should not only grow rapidly to compete for more light, but also improve the defense response to resist the harm of pathogenic microorganisms. How soybeans balance the allocation of resources between growth and defense is unclear. In this paper, we explored the effects of different light intensity on the physiological and biochemical indexes of SMV-infected soybeans, and compared the changes of defense mechanism of SMV-infected soybeans under different light intensities by RNA-seq sequencing method. The results of this study will provide a basis for the study of SMV resistance in intercropping soybean.

## 2 Methodology

### 2.1 Plant material, virus inoculation, and light treatment

Soybean seeds (Nannong 1138-2, a SMV susceptible variety) were kindly provided by Dr. Kai Li from Nanjing Agricultural University in China. The SMV isolate (YA87) was collected from the field soybean plants in Sichuan Province, China. The bean Phaseolus vulgaris cv. Topcrop was used for local-lesion purification of SMV, and then the virus was propagated on the soybean cv. Nannong 1138-2 (Zhang et al., 2019). Soybean seeds were surface-sterilized and sown in a mixed matrix containing PINDSTRUP organic soil (Pindstrup Mosebrug A/S, Ryomgaard, Denmark) and vermiculite (v:v, 4:1) in an artificial climate chamber with 25 °C/22 °C day/night temperature, 60% relative humidity and 14 h/10 h of photoperiod.

Select soybean seedlings with the same growth and inoculate them with the virus (Shang et al., 2011). Leave it in the dark for 12 hours for light treatment. They were treated with hard light, normal light, the shade and the dark. For each light condition, three soybean strains were inoculated with virus and three soybean strains were inoculated with virus-free phosphoric acid buffers as controls (**Table 1**). Placed in a culture environment with a temperature of 25°C and a humidity of 60%.

**Table 1:** Four light intensities gradients.

Light intensity	Control	Virus vaccination
8.11μmol/m^2^s	DC: Dark/control	DS: Dark/SMV
121.63μmol/m^2^s	LC: Low light/control	LS: Low light/SMV
349.73μmol/m^2^s	NC: Normal light/control	NS: Low light/SMV
522.97μmol/m^2^s	HC: Hard light/control	HS: Hard light/SMV

### 2.2 Sample collection and sequencing

Previous studies have shown that larger changes in transcriptional levels occurred in soybeans infected SMV at 10 dpi (Zhang et al., 2019). Therefore, we collected the V2 leaves (the second trifoliolate leaf, newly grown) of soybean plants after treatment for 10 dpi. Total RNA was extracted using phenol-chloroform-isoamyl alcohol and lithium chloride, washed by using 70% ethanol, and finally checked by Agilent 2100 Bioanalyzer to ensure RIN number > 7.0. After the samples were tested, cDNA libraries were constructed and paired-end sequencing was performed based on the Illumina HiSeq 2500 platform at Nuohezhiyuan BioInformation Technology Co., LTD. (Tianjin, China). Three biological replicates were set up for each treatment and a total of 24 independent samples were used for RNA-Seq.

### 2.3 Read alignment and expression analysis

The reads number, Q30, N (%), Q20 (%), and Q30 (%) of raw data was counted. After removing reads containing sequencing adapters and reads of low quality, the clean data were mapped to the reference genome of Glycine max (Glyma2.0) using Bowtie2 and Tophat2. The reads mapped to exon region were also counted. HTSeq (Version 0.11) was used to calculate the read count mapped to each gene as the expression level of the gene at the initial stage. Gene expression levels were normalized using the RPKM (reads per kb per million reads) method. Differential expression analysis between treatments was identified by DESeq2 with screening parameters of log_2_FC (fold change) > 1 and p-adj (adjusted p-value) < 0.05 (Zhang et al., 2019).

### 2.4 Functional enrichment analysis of DEGs

The latest genomic reference information of Glycine max was obtained from the Soybase (www.soybase.org), including Gene Ontology (GO) annotations for each gene. The Kyoto Encyclopedia of Genes and Genomes (KEGG) annotations was obtained from the KEGG database. A hypergeometric test was used to find out the GO terms and KEGG pathways that was significantly enriched by DEGs. The enrichment analyses of GO and KEGG were performed using the OmicShare online website (www.omicshare.com/tools).

### 2.5 Validation of gene expression by qRT-PCR

To verify the accuracy and reproducibility of the RNA-Seq data, RT-qPCR assays were conducted with gene specific primers. Total RNA from the samples was extracted. Reverse transcription was performed using 5× All-In-One RT Master Mix kit (AccuRT Genomic DNA Removal Kit, ABM, Vancouver, Canada). In addition, 2×RealStar Fast SYBR qPCR Mix (GenStar, Beijing, China) was used and Eppendorf Mastercycler ep realplex (Eppendorf, Hamburg, Germany) instrument was used for the RT-qPCR experiment. Each treatment contained three independent biological replicates and three technical replicates. The expression level of soybean β-actin gene was used as an internal reference. The fold change value of gene expression was calculated using the 2−ΔΔCt method. The sequences of specific primers were listed in **Table S1**.

### 2.6 Statistical analysis

The one‐way ANOVA model was used for analyses of the error in IBM SPSS Statistic 27, and the average value was taken. The significance was judged by the new complex range method (Duncan’s method) at p < 0.01.

## 3 Results and discussion

### 3.1 Phenotype and virus content of infected soybean

**Figure 1:**
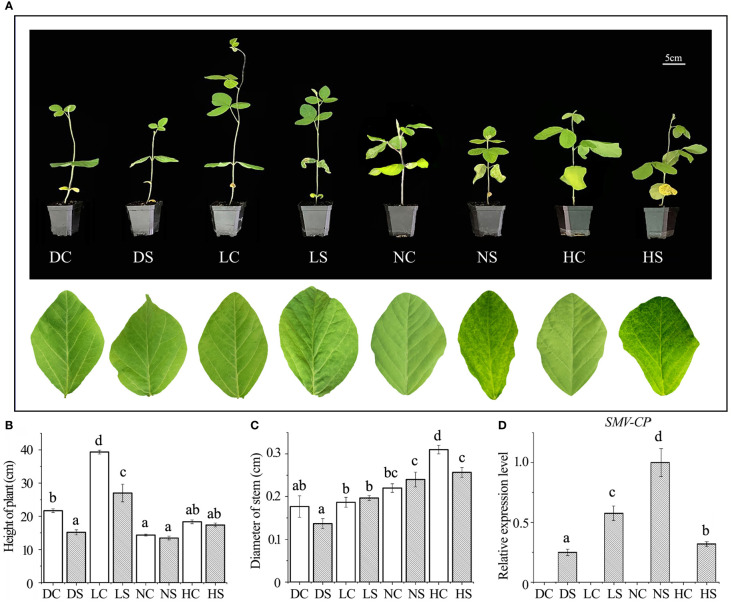
Phenotype and virus content of soybean infected with SMV under different light intensities. **(A)** Phenotype of SMV-infected soybean under different light intensities; **(B)** Plant height of soybean under different treatments; **(C)** Soybean stem diameter under different treatments; **(D)** Quantitative detection of SMV-CP content by qPCR. DC, dark+control; DS, dark+SMV; LC, low light+control; LS, low light+SMV; NC, normal light+control; NS, normal light+SMV; HC, hard light+control; HS, hard light+SMV; Data are expressed as mean ± standard deviation. Values with different letters in a column differ significantly (p < 0.05).

After inoculation with SMV, soybean leaves under hard and normal light showed obvious mosaic symptoms. Dwarfing was observed in all SMV-inoculated soybeans compared to the controls (**Figure 1A**). Compared with the control group, the plant height of infected soybean was not decreased significantly under normal light and hard light, but the pitch spacing was shortened. In the dark and the shade, the plant height of infected soybean decreased significantly compared with the control group. In the shade and under normal light, the stem diameter of infected soybeans increased slightly compared to the control group due to the virus infection, which caused significant shrinkage. However, the stem diameter of hard light and dark light was smaller than that of control group (**Figures 1B, C**). Compared with groups in the dark, the shade and under hard light, the accumulation of virus increases under normal light (**Figure 1D**).

**Figure 2:**
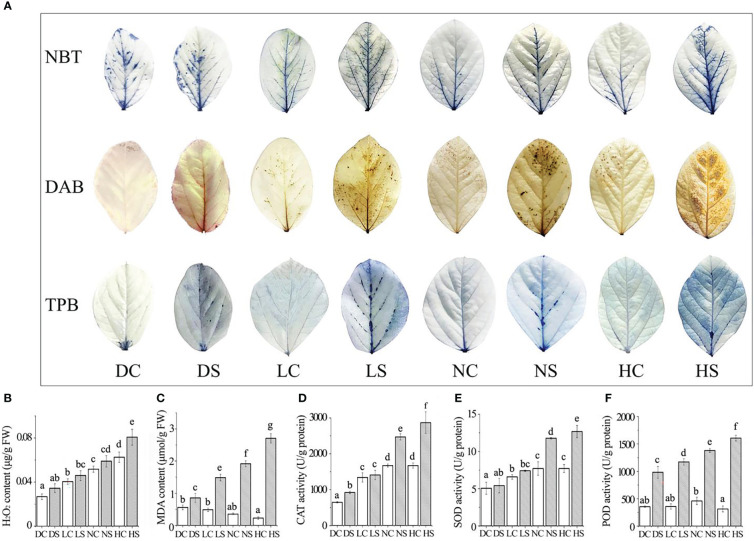
Staining results, membrane lipid peroxidation damage index and the enzyme activity were determined. **(A)** NBT, DAB and TPB staining results; **(B)** hydrogen peroxide; **(C)** malondialdehyde content; **(D)** CAT enzyme activity; **(E)** SOD enzyme activity; **(F)** POD enzyme activity. DC, dark+control; DS, dark+SMV; LC, low light+control; LS, low light+SMV; NC, normal light+control; NS, normal light+SMV; HC, hard light+control; HS, hard light+SMV; Data are expressed as mean ± standard deviation. Values with different letters in a column differ significantly (p < 0.05).

Viral infection often causes plant dwarfing (Shang et al., 2011). SMV inoculation resulted in dwarfing of soybean plants. SMV-infected soybeans were dwarfed compared to controls even when the main stem of the soybeans in the shade and the dark was elongated. Our previous study found that shade resulted in decreased defense ability of soybean (Zhang et al., 2019). The down-regulation of defense ability reduced the inflammatory response of soybean, which was conducive to improving the tolerance to the pathogen. At the same time, more resources can be allocated to the growth of soybean. Our previous studies have shown that reactive oxygen species can promote viral infection (Shang et al., 2009; 2010; 2011; 2019; Tang et al., 2019). Compared with groups under normal and hard light, reactive oxygen species in soybean decreased in the shade and the dark (**Figure 2A**), leading to a decrease in virus content (**Figure 1D**).

Plant growth is regulated by auxin (IAA) and gibberellin (GA). The levels of gibberellin and auxin were detected in stems and leaves of soybean (**Figure S1**). The contents of gibberellin and auxin in soybean decreased after virus infection compared with the controls. Compared with control, auxin and gibberellin in stem and leaf of soybean decreased dramatically under normal light. Under normal light, soybeans have more resources allocated for defense. Compared with control, auxin and gibberellin in stem and leaf of soybean decreased slightly in the shade and the dark. This suggested that plants with limited resources are more likely to allocate resources to vegetative growth and moderate resistance to viruses. Compared with control, auxin and gibberellin in stems and leaves of soybean also decreased slightly under high light, which may be related to photo-inhibition.

### 3.2 Histochemical staining and membrane damage index determination

NBT and DAB staining showed the highest content of superoxide and hydrogen peroxide in soybean inoculated with SMV in the dark. With the increase of light, the content of superoxide and hydrogen peroxide increased gradually compared with the control group. Trypan blue staining showed more severe necrosis in SMV-inoculated soybeans than in healthy plants (**Figure 2A**). Compared with the healthy control, SMV infection induced the accumulation of hydrogen peroxide. As the light intensity increased, so did the hydrogen peroxide content (**Figure 2B**). But the accumulation of hydrogen peroxide in soybean plants under darkness was higher. Malondialdehyde (MDA) content increased with the increase of light exposure compared with the control group. SMV infection induced more MDA accumulation (**Figure 2C**).

Light increased the content of reactive oxygen species (ROS) (Vuleta and Manita, 2016). This was confirmed by soybean leaf staining results. ROS outbreaks activate defense signaling pathways to defend against pathogen attacks (Li et al., 2017). The accumulation of reactive oxygen species causes damage to cell membranes (Shang et al., 2011), thus increasing malondialdehyde levels. Hard light damages the photosynthetic system of leaves, and leaves suffer serious oxidative damage (Chang et al., 2013).

### 3.3 Determination of enzyme activity

The activity of antioxidant enzymes increased in SMV-infected soybeans compared to the control. The contents of catalase, superoxide dismutase and peroxidase showed the same trend (**Figures 2D–F**). The accumulation of reactive oxygen species activated the antioxidant enzyme system (Shang et al., 2019). Compared with the control group, soybean antioxidant enzyme activity increased significantly more under hard light and normal light than in the shade and the dark. This is because light stimulates cells to produce more reactive oxygen species (ROS) during disease resistance (**Figure 2B**), thus activating the activity of the antioxidant enzyme system.

### 3.4 Statistics of differential genes

**Figure 3:**
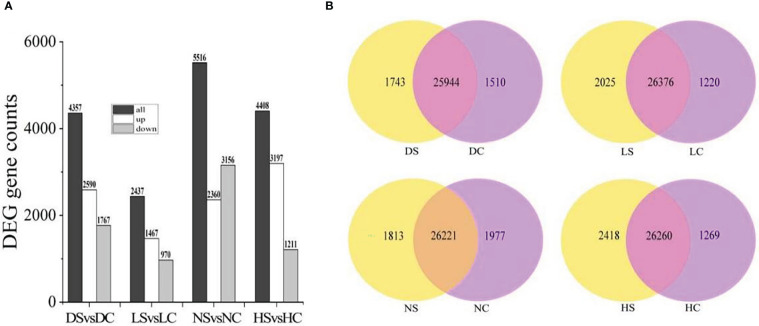
Differential gene statistics. **(A)** Statistics of differential genes between infected SMV and control under different light intensities; **(B)** Overlap analysis of differential genes in the dark, in the shade, under normal light, and under hard light. DC, dark+control; DS, dark+SMV; LC, low light+control; LS, low light+SMV; NC, normal light+control; NS, normal light+SMV; HC, hard light+control; HS, hard light+SMV.

Compared with the healthy control, 2590 genes were up-regulated and 1767 genes were down-regulated in the dark soybean infected with SMV. In the shade, 1467 genes in soybean were up-regulated and 970 genes were down-regulated. Under normal light, 2360 genes were up-regulated and 3156 genes were down-regulated. There were 3197 up-regulated genes and 1211 down-regulated genes in soybean under hard light. Soybean under normal light had the most differentially expressed genes and the most positive response to SMV (**Figure 3A**).

There were 25,944 gene overlaps between SMV-inoculated soybean and healthy control in the dark, 26,376 gene overlaps between SMV-inoculated soybean and healthy control in the shade, 26,221 gene overlaps between SMV-inoculated soybean and healthy control under the normal light. There were 26260 gene overlaps between SMV-inoculated soybean and healthy control under the hard light (**Figure 3B**).

### 3.5 GO function enrichment analysis of DEGs

**Figure 4:**
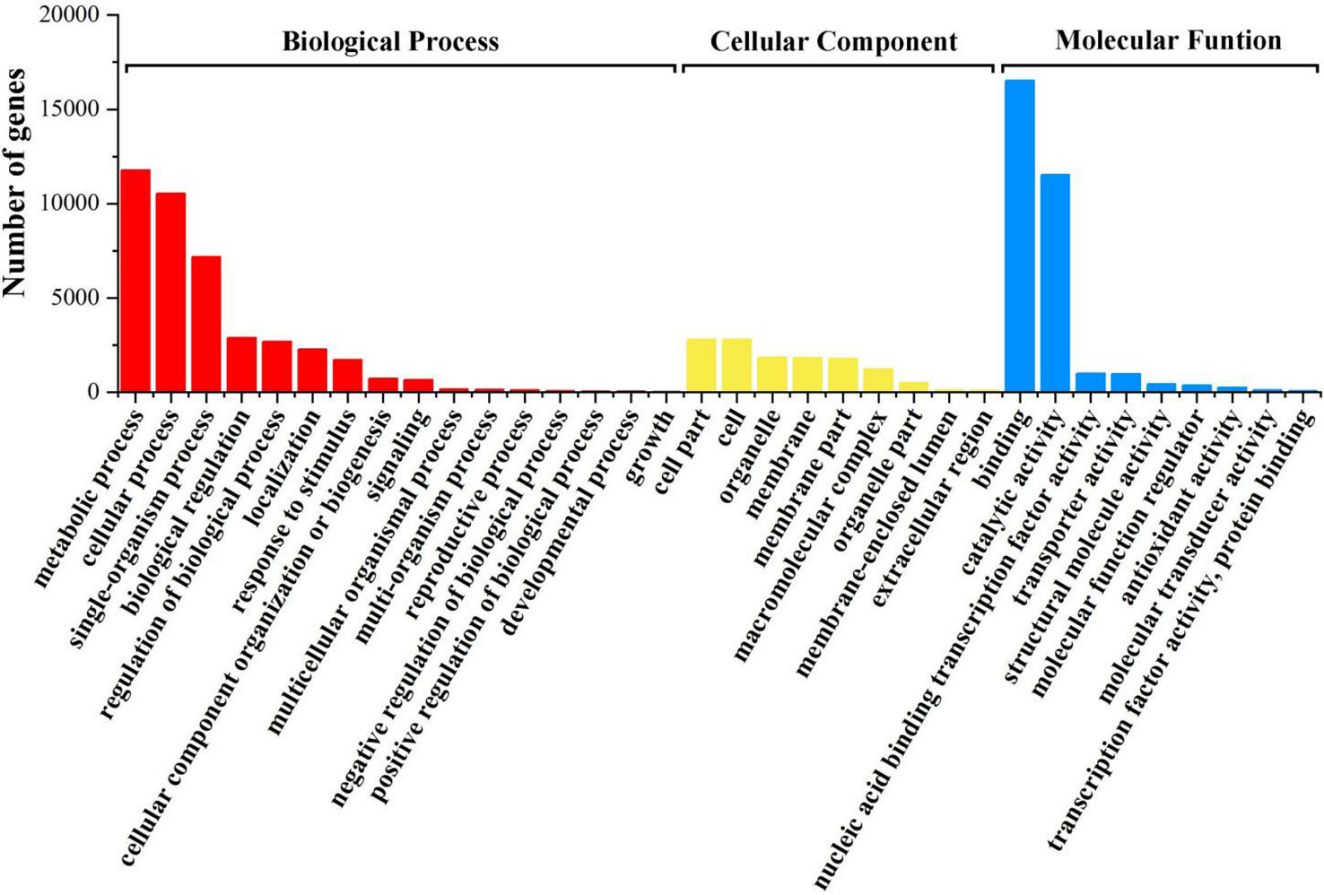
GO functional enrichment analysis of differential genes in total.

Gene Ontology (GO) enrichment analysis was used to determine the functional classification of DEGs between different treatments. Genes were divided into three categories: Biological process, molecular function, and cellular component. When looking at biological processes, the most genes are differentially expressed in cellular processes, metabolic processes and single-organism processes. Among cell components, the most differentially expressed genes were found in the synthesis of the cell wall and the macromolecule complex. In the molecular function (MF), binding and catalytic activity accounted for the highest proportion (**Figure 4**).

**Figure 5:**
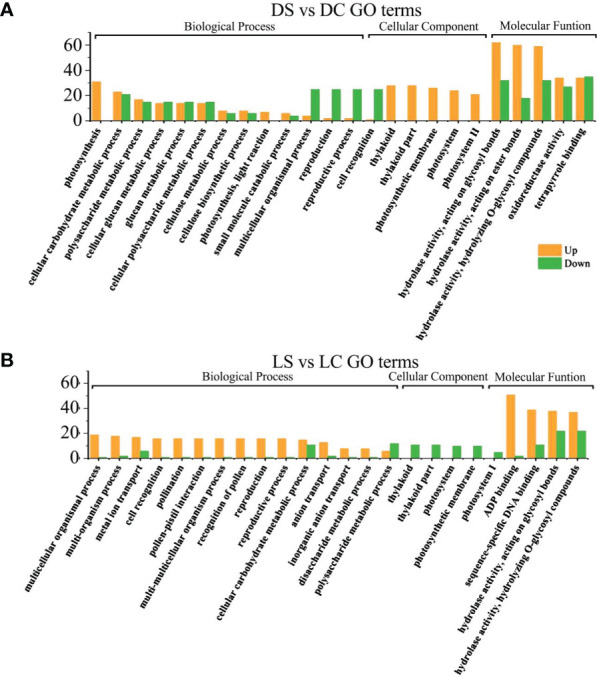
GO functional enrichment analysis of differential genes between infected SMV and control in the dark and the shade. **(A)** GO functional enrichment analysis of differential genes in the dark; **(B)** GO functional enrichment analysis of differential genes in the shade. DC, dark+control; DS, dark+SMV; LC, low light+control; LS, low light+SMV.

In the dark and the shade, differential genes were enriched in photosynthesis and growth and development regulation in infected soybeans (**Figures 5A, B**). In the dark, photosynthesis, cellular carbohydrate metabolic process, multicellular organism process, thylakoid in cellular components, the thylakoid part, photosystem II, photosystem II oxygen evolving complex, hydrolase activity, photosynthetic membrane, Thylakoid Part, Photosystem II oxygen evolving complex, the hydrolase activity, oxidoreductase activity, carbohydrate phosphatase activity and other pathways have the most differentially expressed genes. In the shade, sulfur compound transport, multicellular organism process, floral organ development, thylakoid, photosystem I, photosynthetic membrane, ADP binding, pattern binding, polysaccharide binding in molecular function, hydrolase activity pathway had the most genes.

**Figure 6:**
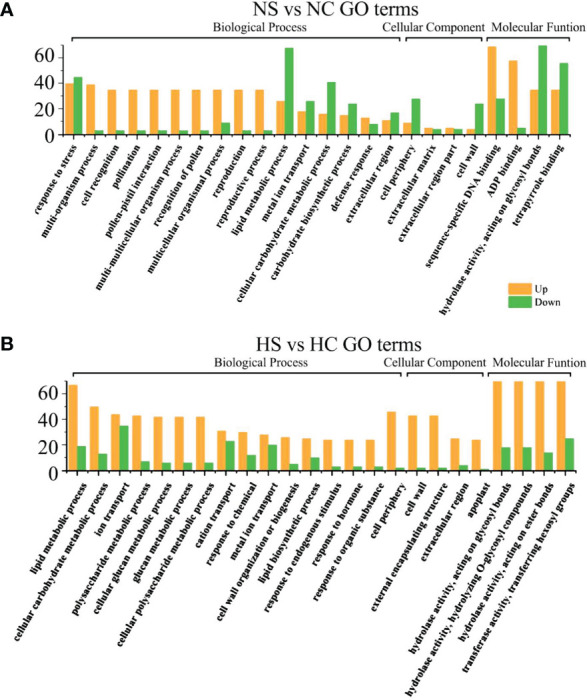
GO functional enrichment analysis of differential genes between infected SMV and control under normal light and hard light. **(A)** GO function enrichment analysis of differential genes under normal light; **(B)** GO function enrichment analysis of differential genes under hard light. NC, normal light+control; NS, normal light+SMV; HC, hard light+control; HS, hard light+SMV.

Under sufficient light, differential genes enriched in cell wall components, cell recognition function and disease resistance in infected soybeans (**Figures 6A, B**). Under normal light, multi-organism process, cell recognition, extracellular region, the cell wall, the hydrolase activity, acting on glycosyl bonds, copper ion binding, hydrolase activity, cellulose synthase activity, acting on glycosyl bonds, copper ion binding, the hydrolase activity, cellulose synthase activity, transferase activity, oxidoreductase activity, cellular metabolic process, cellular glucan metabolic process, glucan metabolic process. Under hard light, cellular metabolic metabolic process, cellular glucan metabolic process, cellular metabolic process, cell wall synthesis, cellulose metabolic process, cell periphery, cellular metabolic process, cell periphery, the cell wall, hydrolase activity, copper ion binding, glucosyltransferase activity, enzyme inhibitor activity, the hydrolase activity, heme binding, tetrapyrrole binding pathway had the most genes.

Under normal light and hard light, most of the DEGs in infected soybean were mainly expressed above regulation. Genes related to glucose metabolism, hydrolase activity, antioxidant enzyme activity, cellulose metabolism, cell wall synthesis and other pathways related to disease resistance were significantly up-regulated. When light is insufficient, plants allocate major resources to the regulation of photosynthetic system and growth and development. Previous studies have also shown that the disease resistance of plants decreased in the shade (Zhang et al., 2019). In the dark and the shade, the number of down-regulated genes increased in soybean infected with SMV compared with the controls. (**Figures 5A, B**). Our previous studies have shown that light is crucial for defense activation (Zhang et al., 2019). The results of this experiment further prove that light intensity has a positive regulatory effect on soybean defense response.

### 3.6 KEGG pathway enrichment analysis of DEGs

**Figure 7:**
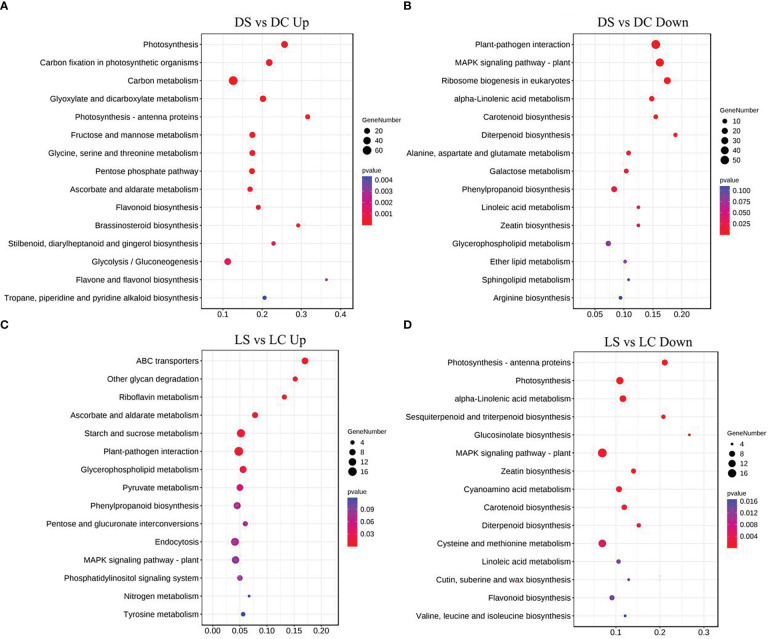
Differential gene KEGG pathway enrichment analysis in soybeans in the dark and the shade. **(A, B)** Up-regulated and down-regulated differential gene KEGG pathway enrichment analysis in the dark; **(C, D)** Up-regulated and down-regulated differential KEGG pathway enrichment analysis in the shade. DC: dark+control; DS, dark+SMV; LC, low light+control; LS, low light+SMV; NC, normal light+control; NS, normal light+SMV; HC, hard light+control; HS, hard light+SMV.

**Figure 8:**
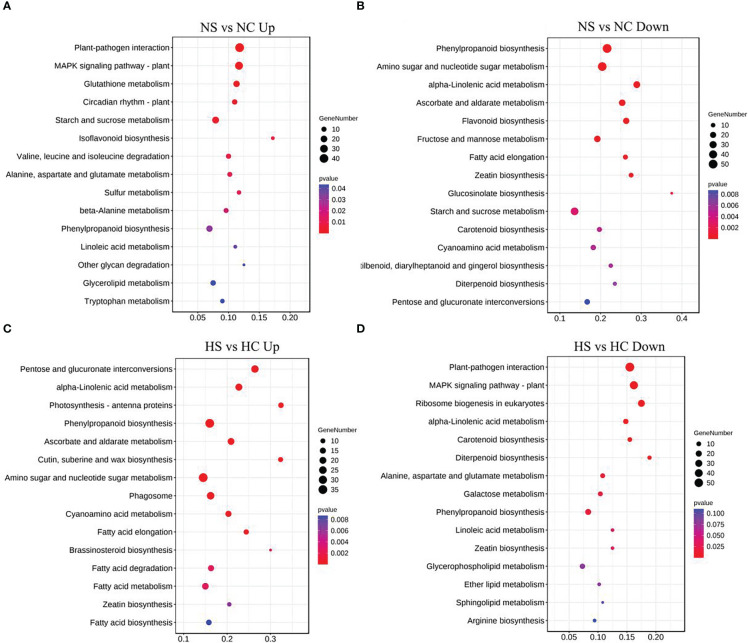
Differential gene KEGG pathway enrichment analysis in soybeans under normal light and hard light. **(A, B)** Up-regulated and down-regulated KEGG pathway enrichment analysis under normal light; **(C, D)** Enrichment analysis of up-regulated and down-regulated differential gene KEGG pathway under the hard light. DC, dark+control; DS, dark+SMV; LC, low light+control; LS, low light+SMV; NC, normal light+control; NS, normal light+SMV; HC, hard light+control; HS, hard light+SMV.

Metabolic pathways of the four light-intensity susceptible soybeans were analyzed, and the top 15 KEGG metabolic pathways with the most differentially expressed genes were shown in **Figures 7** and **8**. Differential genes enriched in carbon metabolism, glycolysis and photosynthesis pathways were most up-regulated in infected soybeans under darkness (**Figure 7A**). The most down-regulated genes were those enriched in plant-pathogen interaction pathway and MAPK signaling pathway (**Figure 7B**). In the shade, the most up-regulated genes in infected soybean were differentially expressed in plant-pathogen interaction pathway, the endocytosis pathway, starch and sucrose metabolic pathway (**Figure 7C**). The most down-regulated genes were MAPK signaling pathway, photosynthetic pathway and cysteine and methionine metabolic pathway (**Figure 7D**). Under normal light, the most up-regulated expression in infected soybeans was concentrated in plant-pathogen interaction pathway, MAPK signaling pathway, glutathione metabolic pathway, starch and sucrose metabolic pathway (**Figure 8A**). The most down-regulated genes were those that were enriched in phenylpropanoid biosynthesis, amino sugar and nucleotide sugar metabolism, ascorbic acid and alnus acid metabolism pathway, and flavonoid metabolism pathway (**Figure 8B**). Under hard light, the most up-regulated genes in infected soybeans were those that were enriched in amino sugar and nucleotide sugar metabolism, phenylpropanoid biosynthesis, pentose and glucuronic acid interconversion, and α-linolenic acid metabolism (**Figure 8C**). The most down-regulated genes were those enriched in MAPK signaling pathway and plant-pathogen interaction pathway (**Figure 8D**). These results suggest that when light intensity is low, plants allocate more resources to vegetative growth. Light intensity can positively regulate the defense regulation of plants, but too hard light intensity will reduce the defense.

### 3.7 DEGs involved in plant-pathogen interaction

Considering the expression levels of soybean related genes under four light intensities, 19 of the most differentially expressed genes were enriched into plant-pathogen interaction pathways. The genes involved in the plant immunity and the pathogen infection constitute the plant-pathogen interaction pathways (Dodds and Rathjen, 2010). Differentially expressed genes mainly included NBS-LRR family genes, WRKY transcription factors, MAPK genes, MYB transcription factors and hormone pathway-related defense genes (**Tables 2**, **3**). WRKY and MYB are transcription factors involved in plant stress resistance under both biological and abiotic stresses (Zhang et al., 2019). WRKY transcription factors act on SA and JA downstream defense genes to play a positive and negative role in regulating disease resistance (Tang et al., 2013). The NBS-LRR class is the largest class of R genes and is mainly responsible for ATP hydrolysis as well as releasing signals. The NBS-LRR can help plants effectively fight against a variety of pathogens, including viruses, bacteria, fungi, nematodes, and insects. Most of these overlapping genes were induced under normal light. Some of those genes induced under hard light. In the dark and the shade, resistance-related genes were down-regulated. When light is insufficient, the defense of soybean is inhibited.

**Table 2:** Overlapped DEGs related to plant-pathogen interactions.

Gene ID	Gene Description	DSvsDC log_2_FC	LSvsLC log_2_FC	NSvsNC log_2_FC	HSvsHC log_2_FC
100820033	NB-ARC domain-containing protein	-1.781	7.878	12.378	-4.265
100803026	YODA MAPKK kinase	1.408	5.308	10.000	0.699
100792603	TMV resistance protein,NB-ARC	1.201	8.398	9.090	2.491
102659638	TMV resistance protein N, TIR domain	-0.233	7.815	7.261	—
100816991	probable WRKY transcription factor 40	—	6.685	7.261	-9.106
100793122	disease resistance protein, NB-ARC	-5.510	6.821	7.154	2.576
100796836	probable WRKY transcription factor 50	0.914	5.560	6.995	-1.522
100810978	probable WRKY transcription factor 40	0.236	3.577	5.889	-7.544
102670495	probable WRKY transcription factor 70	—	2.002	4.167	-7.131
100776837	probable WRKY transcription factor 33	-1.803	-1.171	2.140	-3.506
100792193	probable WRKY transcription factor 70	—	0.617	1.670	-8.377
100306201	Pathogenesis-related protein	—	2.461	1.311	-6.208
100791870	probable WRKY transcription factor 40	-1.612	2.457	0.695	-8.732
100781032	transcription factor MYB14	—	1.495	-1.084	-8.257
100783267	Pathogenesis-related protein	1.242	1.912	-1.992	9.144
100795186	Pathogenesis-related thaumatin protein	2.601	-1.290	-2.542	8.922
778179	MYB transcription factor MYB73	0.714	-3.010	-8.536	1.214
100819665	MAPK/ERK kinase 1	—	-2.429	-9.629	7.925
100775603	transcription factor MYB14	0.675	-2.639	-10.587	3.878

**Table 3:** DEGs involved in plant defense hormone signaling.

Gene ID	Gene Description	DSvsD log_2_FC	LSvsL log_2_FC	NSvsN log_2_FC	HSvsH log_2_FC	Pathway
100527824	NPR1 interacting protein	0.251	1.559	4.449	-0.339	SA
100797074	NPR1 interacting	-2.459	1.970	3.417	-1.752	
100805261	Protein SUPPRESSOR OF NPR1-1	3.116	1.433	-1.217	1.478	
100814739	Regulatory protein NPR3	0.062	2.179	3.946	0.960	
100807250	pathogenesis-related protein 1	—	4.142	6.477	-2.628	
100805116	pathogenesis-related protein 1	-0.585	0.014	3.543	-1.573	
100527073	pathogenesis-related protein 10	-0.994	0.864	2.036	-1.822	
100808443	Transcription factor TGA1	2.088	1.090	1.905	0.080	
100794268	Transcription factor TGA3	0.212	1.208	1.393	-2.577	
100792358	Jasmonic acid-amido synthetase JAR1	0.244	-1.783	-3.467	2.225	JA
100813472	Jasmonic acid-amido synthetase JAR1	—	-0.426	-2.517	1.979	
100819069	Coronatine-insensitive protein 1	-0.322	0.511	1.311	-0.912	
100793081	Coronatine-insensitive protein 1	0.153	-0.137	1.201	-0.670	
102663214	transcription factor MYC1	-2.641	-0.806	2.284	-2.138	
100790854	transcription factor MYC2	-0.359	-2.319	-1.502	0.947	
100804965	transcription factor MYC2	-1.217	-2.593	-4.036	2.718	
100795733	transcription factor MYC2	0.020	-2.038	-4.168	3.759	
100819971	ethylene-responsive transcription ERF023	2.665	-0.355	-7.755	7.046	ET
100101914	ethylene-responsive transcription ERF034	-1.016	-1.123	-6.713	3.327	
100811328	ethylene-responsive transcription ERF054	-5.865	4.920	-5.856	7.152	
100778793	ethylene-responsive transcription ERF027	-6.274	-1.342	-5.028	1.974	
100785936	ethylene-responsive transcription factor 1B	-2.309	1.014	4.614	-4.511	
100804481	ethylene-responsive transcription factor 1B	-2.064	-1.140	2.791	-3.135	
100790598	ethylene-responsive transcription factor 1B	-2.368	-1.295	1.973	-1.994	
100801528	ethylene-responsive transcription ERF010	-0.672	-0.367	1.444	-2.449	
100786182	EIN3-binding F-box protein	-1.204	-0.460	1.240	-0.040	
100805418	EIN3-binding F-box protein	-1.356	-0.292	1.214	-0.293	

### 3.8 DEGs involved in plant hormone signal transduction

The differentially expressed genes involved in the three plant hormone signaling pathways of salicylic acid, jasmonic acid and ethylene are shown in **Table 2**. The virus is a live trophic pathogen (Shang et al., 2011). Soybean can resist the virus infection mainly by up-regulating salicylic acid signaling pathway genes. Normal light significantly activated salicylic acid pathway defense genes. In all treatments, defense genes of jasmonic acid pathway were down-regulated in infected soybeans. Under normal light and hard light, some defense genes of ethylene signaling pathway were significantly up-regulated and some were significantly down-regulated in infected soybeans.

### 3.9 Validation of RNA-Seq data by qRT-PCR

**Figure 9:**
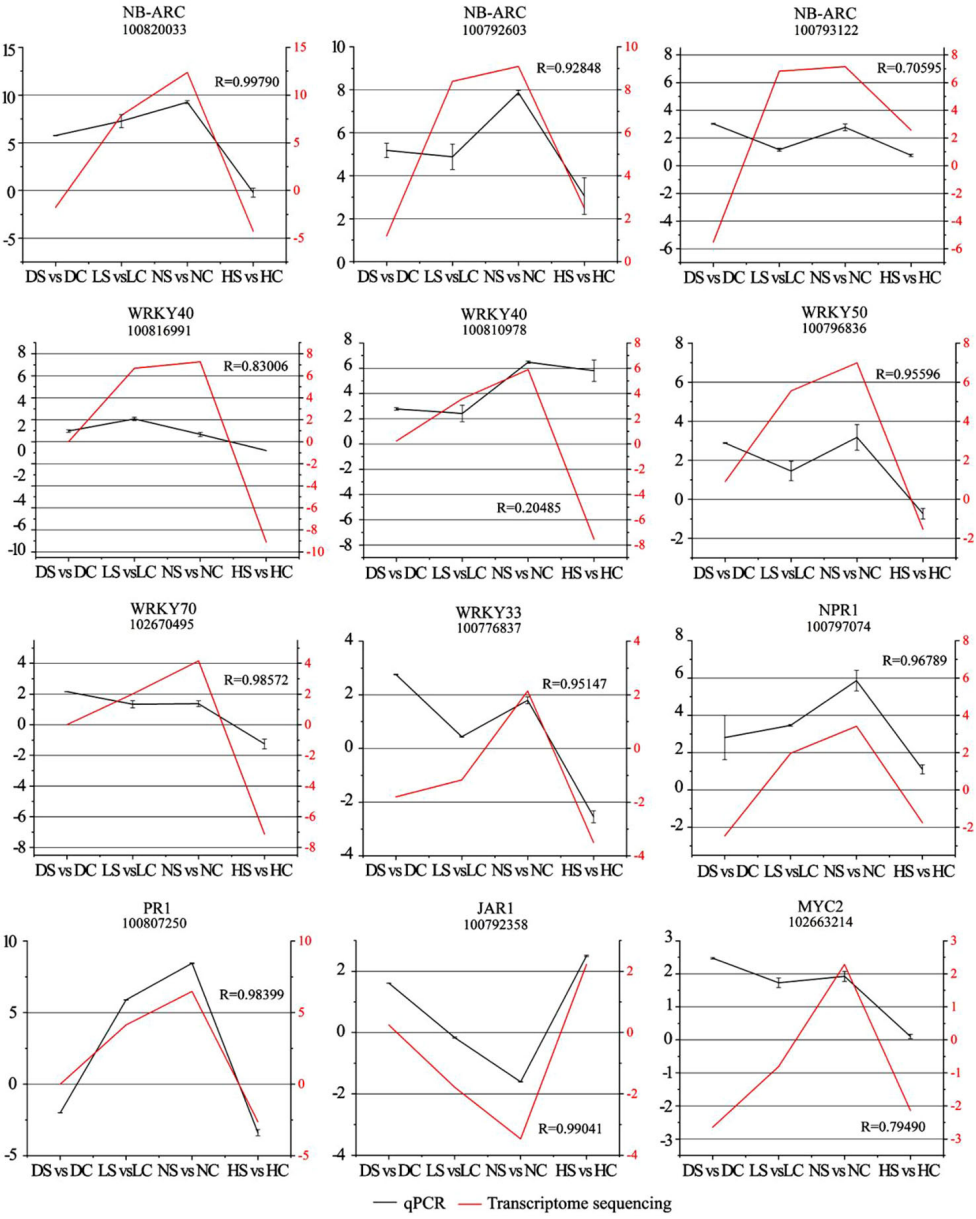
Comparison analysis of resistance-related highly differentially expressed genes by RT-qPCR and transcriptome sequencing. Note: Left vertical axis coordinate is relative expression level of RT-qPCR (black); right vertical axis coordinate is RPKM of RNA-Seq (red). Ordinate axis is the correlation coefficients between RT-qPCR and RNA-seq. DC, dark+control; DS, dark+SMV; LC, low light+control; LS, low light+SMV; NC, normal light+control; NS, normal light+SMV; HC, hard light+control; HS, hard light+SMV.

To further confirm the gene expression pattern obtained from RNA-Seq, 15 DEGs were selected for RT-qPCR, including NB-ARC (100820033, 100792603, 100793122), WRKY40 (100816991, 100810978), WRKY50 (100796836), WRKY70 (102670495), WRKY33 (100776837), NPR1 (100797074), PR1 (100807250), JAR1 (100792358), MYC2 (102663214), and the transcriptome sequencing results were fitted and analyzed (**Figure 9**).

Most of the gene expressions were sensitive to changes in light intensity and the virus invasion and were significantly induced under normal light. However, hard light down-regulated the expression of the above genes. The expression of these genes in soybean was inhibited in the shade and the dark. It shows that suitable light is necessary to activation of the defense in soybean. When the growth of soybean was weak under low light, high resistance will be detrimental to the plant (Shang et al., 2011). Light induces excess heat energy in soybean during photosynthesis (Shang et al., 2019). In order to reduce soybean consumption under hard light, some resistance genes were down-regulated. In order to adapt to the change of light intensity, soybean balanced allocation of resources between growth and defense through a series regulation of gene expression. In general, gene expression patterns displayed by RT-qPCR and RNA Seq tend to be consistent.

### 3.10 Quantitative analysis of differentially expressed genes related to hormone pathways

**Figure 10:**
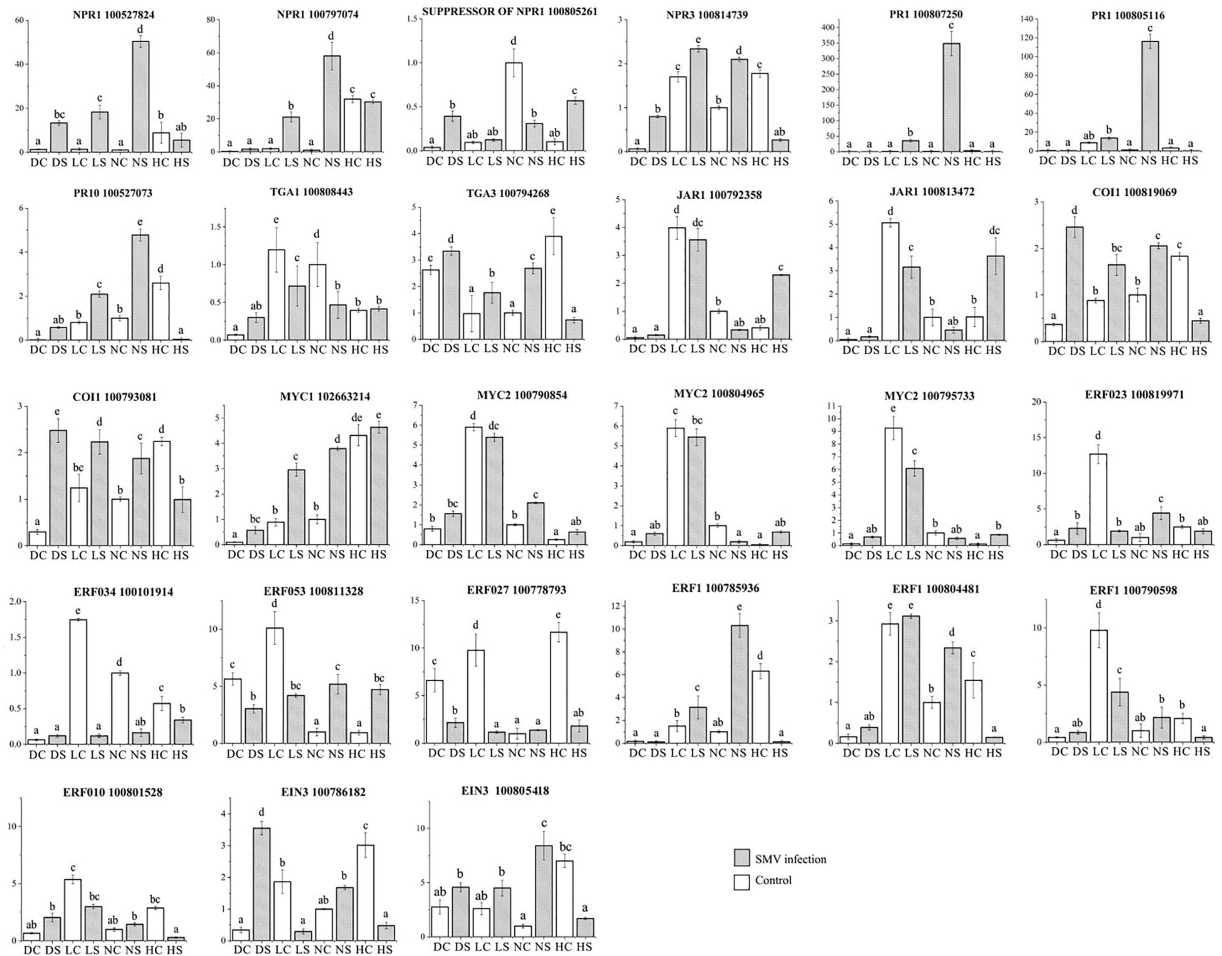
Quantitative analysis of differentially expressed genes related to hormone pathways by qPCR. DC: dark+control; DS: dark+SMV; LC: low light+control; LS: low light+SMV; NC: normal light+control; NS: normal light+SMV; HC: hard light+control; HS: hard light+SMV. Data are expressed as mean ± standard deviation. Values with different letters in a column differ significantly (p < 0.05).

Quantitative analysis of defense gene expression of salicylic acid pathway showed that NPR1 expression of infected soybean was up-regulated under normal light. Compared with the control, the expression of NPR1 gene in infected soybean was down-regulated under hard light, in the shade and the dark (**Figure 10**). At the same time, the expression level of NPR1 suppressor gene was opposite to that of NPR1 gene under different light intensities. The expression of NPR3 gene in infected soybean was down-regulated in the dark and under hard light compared with the control. The expression of NPR3 in infected soybean was up-regulated in the shade and under normal light compared with the control. Under normal light, PR1 and PR10 genes were significantly up-regulated in infected soybean compared with the control. In the dark and the shade, the above genes were slightly up-regulated. Previous studies have shown that PR family genes are induced by light, but suppressed under hard light (Agrawal et al., 2000; Wu et al., 2020). Under hard light, they significantly down-regulated expression. The expression of TGA1 gene in infected soybean was down-regulated in the shade and under normal light compared with the control. The expression of TGA3 gene in infected soybean was significantly down-regulated under hard light compared with the control. However, the expression of TGA3 in infected soybean was up-regulated in other treatments compared with the control. TGA gene may be a key regulatory gene for the antagonism between salicylic acid and jasmonic acid (Caarls et al., 2015). This indicates that light is one of the important conditions for activating salicylic acid defense reaction, which is consistent with previous studies (Backer et al., 2015). Hard light inhibited the expression of salicylic acid pathway defense genes in plants. The increase of light intensity will aggravate the mosaic symptoms of leaves. The severity of symptoms is proportional to the virus content (**Figure 1**). Hard light will cause light suppression and damage, and lack of light will make the plant grow weak. Therefore, down-regulating the expression of defense genes can reduce the inflammatory response, which is a self-protection mechanism to balance defense and growth of plants.

The determination of hormone content showed that the removal of salicylic acid played an important role in the antiviral process of soybean. Compared with the control, the salicylic acid content in infected soybeans increased significantly under normal light and hard light. Salicylic acid increased slightly in infected soybeans in the dark and the shade compared with the control (**Figure S2**). Levels of jasmonic acid and ethylene decreased in infected soybeans compared with the control, but increased under hard light. It indicated that soybean could resist the virus infection mainly by increasing salicylic acid content. But when salicylic acid concentration was too high, the contents of ethylene and jasmonic acid would increase and antagonize the salicylic acid pathway. In the shade, ethylene content in infected soybean increased significantly compared with control. This is more conducive to the downregulation of resistance and inflammatory response.

Quantitative analysis of defense gene expression of jasmonic acid pathway showed that JAR1 gene was down-regulated in infected soybean in the shade and under normal light. JAR1 gene was up-regulated in infected soybean under hard light. COI1 expression in soybean was up-regulated in the dark and the shade, and down-regulated under hard light. The expression of MYC2 gene was down-regulated in soybeans in the shade and under normal light, and up-regulated in soybeans under hard light. Our previous studies have shown that jasmonic acid pathway and salicylic acid pathway antagonizes each other against the virus infection (Shang et al., 2011).

The expression of ethylene related ERF023, ERF034, ERF053 and ERF027 in infected soybean was significantly down-regulated in the shade and under normal light, and up-regulated under hard light. Under normal light, ERF1 gene expression in infected soybean was significantly up-regulated. ERF1 gene was down-regulated in infected soybean under hard light and in the shade. EIN3 gene was down-regulated in infected soybean in the dark and under normal light. Under hard light, EIN3 expression in infected soybean was significantly down-regulated. Hard light causes EIN3 to break down (Shi et al., 2016).

## 4 Conclusion

In this study, the mechanism of soybean response to *Soybean mosaic virus* infection under different light intensities was analyzed using RNA-seq sequencing technology. The induction of defense genes by light was significant. Soybean fights SMV infection mainly by activating the salicylic acid defense signaling pathway. Oxidative damage caused by increased light intensity promotes viral infection. In order to reduce oxidative damage, some defense genes of infected soybean were down-regulated under hard light. The change of defense-related gene expression was confirmed by real-time quantitative PCR. In order to adapt to the change of light intensity, soybean balanced allocation of resources between growth and defense through a series of gene expression regulation. The results of this study will provide a theoretical basis for the research of SMV resistance in intercropping soybean. In the future, we aim to analyze the response of key resistance genes to light changes and their role in the antiviral process of soybean.

## Data availability statement

The original contributions presented in the study are included in the article/**Supplementary Material**. Further inquiries can be directed to the corresponding author.

## Author contributions

JS designed the study. JS, L-PZ, and C-SH collected the data. JS, X-MY, X-LQ, and J-FY analyzed the data and prepared the first draft. JS reviewed and edited the final draft. J-BD, KL, W-MW, and W-YY revised the manuscript. All authors contributed to the article and approved the submitted version.
