# Supplementary material for: Soybean balanced the growth and defense in response to SMV infection under different light intensities
Source: Front Plant Sci. 2023 Apr 19;14:1150870. doi: 10.3389/fpls.2023.1150870 (PMC10154679; doi:10.3389/fpls.2023.1150870)

Supplementary Material

**Supplementary Table 1** Primers used in experiments

| **Gene ID** | **Primer F（5'-3'）** | **Primer R（5'-3'）** | **Reference** |
| --- | --- | --- | --- |
| SMV-550 | AGCTCGCTTCGTCTGGAAAA | ATCATCACCCACACGCCATT | Zhang et al. |
| Actin | CCATGTTCCCTGGTATTGCTG | GTATTTTCTCTCCGGTGGTGC | Zhao et al. |
| SMV-CP | GCTTGGACCACTTGCT | ACACCCATCTGCTCATC | This study |
| PTRV2 | CAGGCGGTTCTTGTGTGTCAAC | AAGACAATGAGTCGGCCAAACG | Wu et al. |
| 100820033 | GGACAGCACACAGCCTATACC | ACCGACTGAGTTGTCCACAT | This study |
| 100792603 | ATTCATGGACGAGGAACGCC | ACCAACCCAACTCCATAACCTAT | This study |
| 100816991 | AACAGAACGACCGTGGCA | GGCTCTTGAGGGAGTGGA | This study |
| 100796836 | CAACCAGTGGCCTTTTGAGC | ACGTCCACTGCTAGTATTGCT | This study |
| 100810978 | CCGTGAAAAAGAAGGTGCAAA | GCACTTCGAATTGTGGGTGT | This study |
| 102670495 | AAGAACGGGAGGAGGTGTCT | TGCACCTGTAGTAGTTCCTTGAG | This study |
| 100793122 | TTGGATCGTTGCGATCGTCT | GCCTCATGCAGTTCCTGATTC | This study |
| 100776837 | AGGAAACTCAAATGCAAGGAGC | GGCTCGCTCAACGTGTTTTC | This study |
| 100797074 | TGTCAGATCTCAACTCATGCTTA | CGATCGCGAACATCCTTTGTC | This study |
| 100807250 | CATCAAACGCCTCCC | CCACAATGACTAACCCTAA | This study |
| 100792358 | TCACAACTTTCACCGGACTGT | ACTTTGGGGTCCCATTGTGG | This study |
| 102663214 | CCACCACGACAACGGAAAGA | TGAGGGGCCAGAATAGTCGT | This study |
| 100527824 | CCGAACCTTCCTTTG | CCTCAGACCCATCCA | This study |
| 100805261 | ACTCCGTGGTGTCTTCG | GCTCCTTCTTGTGCCTAC | This study |
| 100814739 | GTGTTGATGTCCTGGAGAGGG | GCCGAGCAAATGCAACTCTAT | This study |
| 100805116 | CAAGACTCACCAGCAGA | CAACCGTGTCATCCC | This study |
| 100527073 | CCCAAGGAAATGGTGGTCCT | CACATACTTGGTTTTGTCACCTTCA | This study |
| 100808443 | GAAGGAACCCACATTCCCCA | TTGTCGTAGATGATCAGCCTGG | This study |
| 100794268 | AACAACCTCCGCCTATCGTC | CCTAAGGTGATCTGCCTGGT | This study |
| 100813472 | GCTGTGGTTCCCACTTTCTCT | CACCTGTACAGTCCCGTGAA | This study |
| 100819069 | ATCGGTCGTTATTGCAGGAATTT | TCCTGGATTCTGACGTTGGC | This study |
| 100793081 | CGGTCGTTATTGCAGGAACTTA | AGTTGGGGCAATTTCTGGCT | This study |
| 100790854 | GACTACCTCCATCTAATAACG | CTGCCATCAACCTTGC | This study |
| 100804965 | AACTGAAGGCGAAGATTG | CCAGACCCGCTTTGAT | This study |
| 100795733 | AGGGTCTTAAATCTGCG | AAACCGACTACTATGAACAG | This study |
| 100819971 | CAGCAACCGTCGTTTATGGC | GCATCTCTTCTGCGACAACG | This study |
| 100101914 | CTTTCACCATTACTACTCCCTC | CCTTCATTACTATTGGCTTTCT | This study |
| 100811328 | GGTCCATAATGATAATGTTTCG | GTCTTGGCGGTAAAGTCG | This study |
| 100778793 | GACCCACCTACTTCCTAT | GGATTTCAGTAACCCATTT | This study |
| 100785936 | TCTATGGCATCATCACTTC | CTGCTCCTCTTCTTATTCAC | This study |
| 100804481 | GCACGGAAGGAGAAGTCG | TGGTCGTAAGCCAGAGCC | This study |
| 100790598 | GTCACGGGATGAGGGTGT | TGGTCGTAAGCCAGAGCC | This study |
| 100801528 | GCTTCAGCAATCCTCGTCCT | ACCAGTCCACGACTCCAGTAAT | This study |
| 100786182 | CCCTTGTCAATTACAGTGGTGA | GTACAAGCGACCCAACTCCA | This study |
| 100805418 | CTCTGAGGCTGGTCTGGT | GCGAGCACTGGGCAACTA | This study |

**Supplementary Figure 1** Determination of IAA and GAI content in different treatments.

**
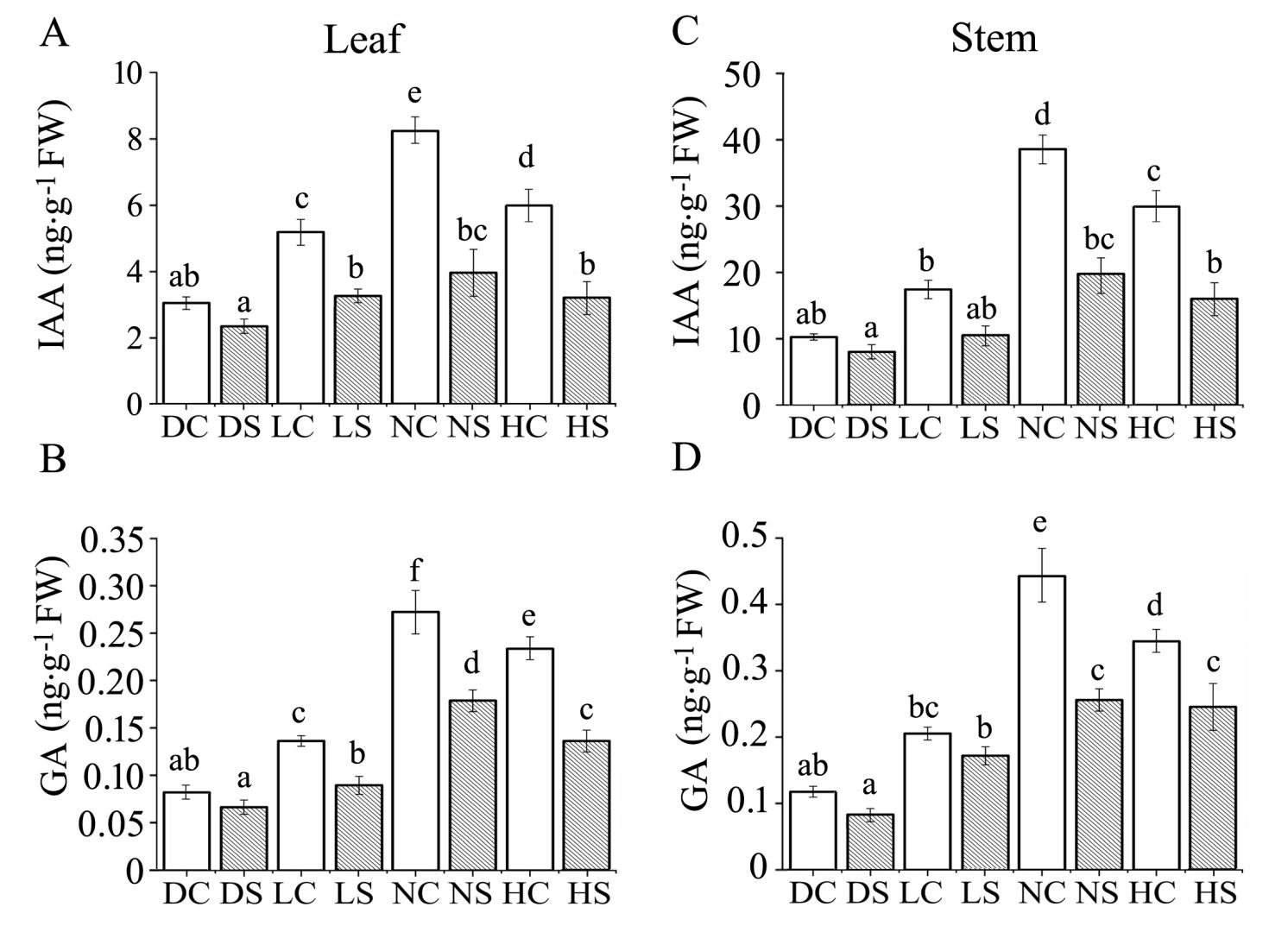
**

**Supplementary Figure 2** Determination of SA, JA and ET content in different treatments.


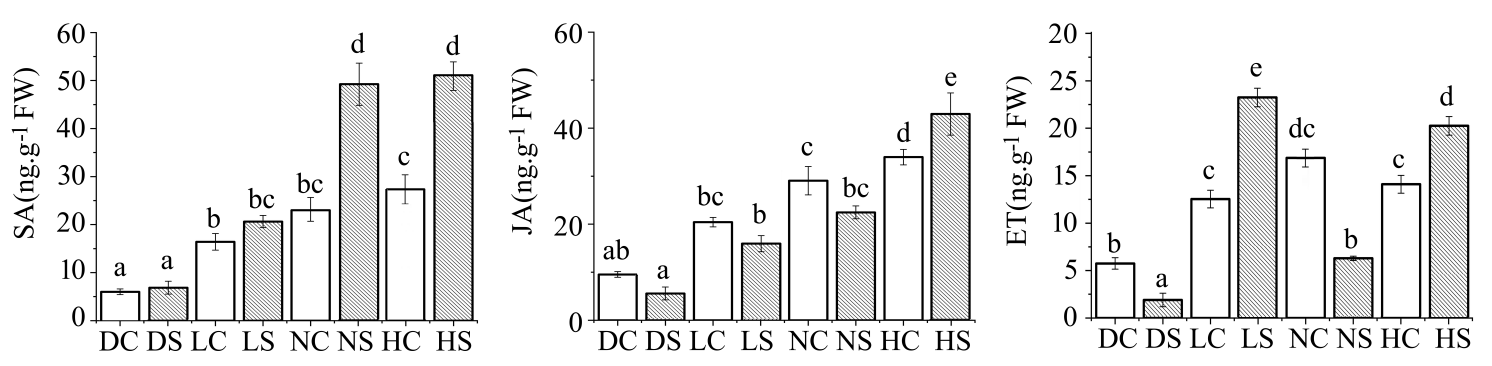

Supplement: Supplementary file 1 [file DataSheet_1.docx]
